# Physical Activity and Cognitive Functioning of Children: A Systematic Review

**DOI:** 10.3390/ijerph15040800

**Published:** 2018-04-19

**Authors:** Ilona Bidzan-Bluma, Małgorzata Lipowska

**Affiliations:** 1 Neuropsychological Diagnostic and Therapy Centre, Chmielna, 80-748 Gdansk, Poland; 2 Institute of Psychology, University of Gdansk, Bażyńskiego 4, 80-309 Gdansk, Poland; malgorzata.lipowska@ug.edu.pl

**Keywords:** cognition, sport, brain, health, childhood

## Abstract

Childhood is an important and sensitive period for cognitive development. There is limited published research regarding the relationship between sports and cognitive functions in children. We present studies that demonstrate the influence of physical activity on health, especially a positive correlation between sports and cognitive functions. The keywords “children, cognition, cognitive function, physical activity, and brain” were searched for using PsycInfo, Medline, and Google Scholar, with publication dates ranging from January 2000 to November 2017. Of the 617 results, 58 articles strictly connected to the main topics of physical activity and cognitive functioning were then reviewed. The areas of attention, thinking, language, learning, and memory were analyzed relative to sports and childhood. Results suggest that engaging in sports in late childhood positively influences cognitive and emotional functions. There is a paucity of publications that investigate the impact of sports on pre-adolescents’ cognitive functions, or explore which cognitive functions are developed by which sporting disciplines. Such knowledge would be useful in developing training programs for pre-adolescents, aimed at improving cognitive functions that may guide both researchers and practitioners relative to the wide range of benefits that result from physical activity.

## 1. Introduction

In recent years, there has been a shift in the lifestyles of various age-groups, including children, especially in their late childhood. Unlike children a few decades ago, children today are leading increasingly sedentary lifestyles that involve time spent on computers and watching TV. This lifestyle leads them to neglect the physical activity that has been typical to this developmental period [1,2]. In times when children are less active, the significance of studies on the positive impact of sport on physical health, mental health, and cognitive functioning, is critical [3,4,5,6,7]. The aim of such research is to raise awareness of the gravity of the problem, as well as to create a holistic health program that promotes being physically active in different contexts, including with family and in school [8].

Limited physical activity, or often a complete lack thereof, leads to various health problems, including posture problems (such as idiopathic scoliosis), somatic conditions, being overweight and obese, problems with circulation, and even premature death [6,9,10,11,12]. There is increasing empirical evidence of a relationship between a lack of physical activity and mental health measures. For instance, research suggests that overweight adolescents who do not practice sports are more prone to risk behaviors, including suicide attempts and addiction to both alcohol and illicit drugs [13,14].

The guidelines referring to physical activity in a report from the National Association for Sport and Physical Education [2] emphasize that children should spend as much time as possible engaging in activities that require physical movement. The World Health Organization and Fonds Gesundes Osterreich [15] further specify guidelines indicating that children should devote at least 60 min a day to physical activity (such as going to school on foot, walking up-stairs, and cycling). Moreover, children should strengthen their muscles and bones with strength training at least two to three times a week [16]. The argument has been made that children who do not do physical exercise will never fully develop their genetic potential in terms of motor skills [17].

**Figure 1:**
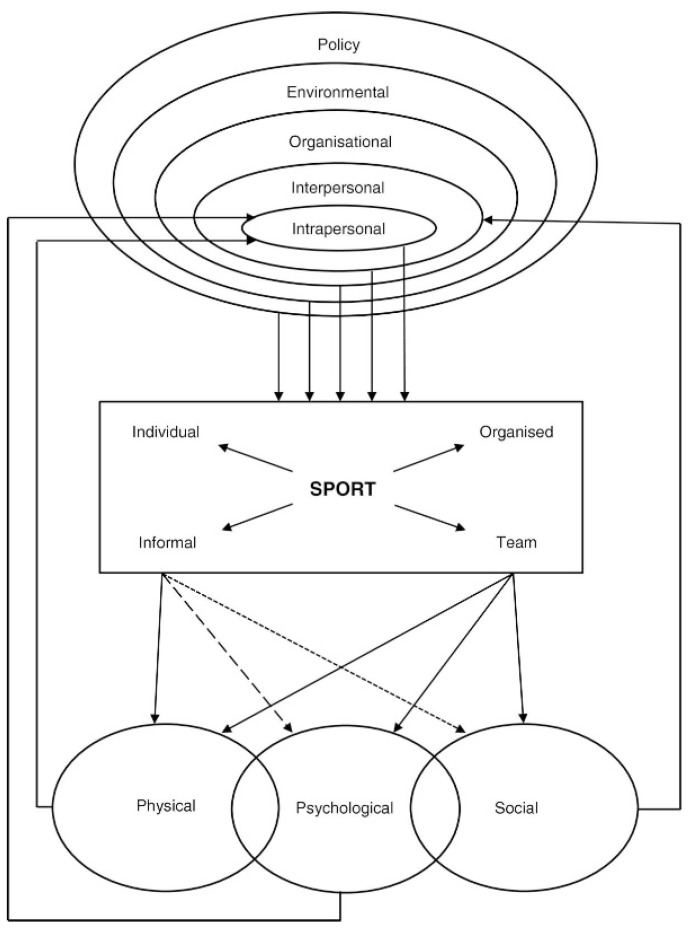
A conceptual model of Health through Sport. Source: Eime, et al. [22].

It has been shown that engaging in sports is a protective factor against somatic illnesses and pathological behaviors [18,19]. Sport provides an equilibrium between group demands and individual demands, between aggressive behaviors and self-control. It fosters a sense of belonging to a group, and teaches coping with both victory and defeat [20]. Increased susceptibility to risk behaviors among adolescents is associated with a not-yet-mature cognitive-control system, which is responsible for impulses associated with engaging in risk behaviors [21]. Regular physical activity also leads to better circulation and oxygen supply to the brain, an increase in bone and muscle density, and greater tolerance of stress [15]. Figure 1 [22] presents the relation between policy, environment, organizational, interpersonal, and intrapersonal factors that affect the undertaking of various types of sports activities, and the physical, psychological, and social benefits brought about by doing sport.

Although it is evident that physical activity is related to physical and mental health [23], the relation between physical activity and cognitive functioning requires further study. Cognitive functions include: memory, attention, visual-spatial, and executive functions, while complex cognitive processes include: thinking (abstract, cause and effect, creative thinking, and planning) and language functions [24]. Despite the importance of this issue, few studies are concerned with the relationship between sports and cognitive functioning of children in late childhood [25], and research to date (unfortunately not free of errors in sampling) provides contradictory results regarding the influence of sports on cognitive functions in children. Some report that sports have a positive influence [3,26,27,28,29], particularly on executive functions, which develop intensively in this period [28,30], as well as mention the positive impact of regular and irregular exercises which lead to an increase in the level of oxyhemoglobin that facilitates the operation of executive functions for up to 30 minutes [31]. Others, however, do not confirm this positive influence of physical activity on cognitive functioning [32,33].

## 2. Methods

This review was conducted using Ebsco Information Services (https://www.ebsco.com/, EBSCO Industries, Inc.) to access two electronic databases (PsycInfo and Medline), as well as Google Scholar. The search focused on articles published from January 2000 until November 2017. Keywords applied in the search were: children, cognition, cognitive function, physical activity, and brain. A total of 617 articles met these original criteria. Articles included some systematic reviews, but were mostly original research. These articles were further assessed, and only those that emphasized the disciplines related to the research topic (psychology, sport, medicine) were included in the final sample. A total of 58 articles were then reviewed, given that many of the articles that emerged in the initial search were connected to attention deficit hyperactivity disorder (ADHD) and disability, rather than physical activity and cognition. Such articles were excluded from the sample. Other publications were referenced to introduce the problem and to discuss its background in the neuropsychological context.

## 3. Results

The following subtopics were examined in the articles that emerged in our review: attention, thinking, language, learning, and memory. Below, we highlight some of the most important findings relative to each of the subtopics, followed by a model that outlines the influence of sport on physical, mental, and social resources.

### 3.1. Attention

The ability to focus attention is improved among children who participate in physical activities [34,35,36]. Cross-sectional studies suggest that with regards to cognitive flexibility and operational memory, the regularity and intensity of physical activity in children aged 13–14 years positively affects their ability to focus attention on a given task [37]. This effect is especially noticeable after the third hour of classroom lessons; the time at which processes related to attention and focus on a given task tend to deteriorate. Students who regularly do sports are also calmer during lessons [15]. Some researchers indicate the lack of connection between mental activity and shifting attention or flexibility of attention. They also emphasize that there is a significant deterioration of these functions among individuals who spend too much time in front of the computer screen and playing too many computer games, as defined by self-reports and objective measures [38]. They also stress that children who do some form of sport (e.g., karate) function better than those who prefer passive activity. This result is observed through better speed times and visual selective attention than in the case of children with a sedentary lifestyle [39].

Studies have also been conducted that focus on physical activity outside of class-time or school settings. These studies revealed that physical activity in non-school contexts enhances selective attention, in contrast to passive activities in non-school contexts [40]. Some studies revealed the positive impact of sport, specifically on executive functions [27,28,36], which develop intensively in the period of late childhood [4,28,39]. Research using various kinds of intervention trials confirms the beneficial influence of physical activity on attention. Even a 12 min session of aerobic exercise improved the selective attention of children [41]. Research has also demonstrated the positive impact of exercise, both regular and irregular, which leads to an increase in the level of oxyhemoglobin, facilitating the operation of executive functions for up to 30 min [31]. At the same time, the unfavorable influence of lack of physical activity on cognitive functioning is reported. It has been found that there is a negative impact of time spent in front of the computer screen on executive functions as related to inhibition [36].

It was also found that children who engaged in physical activity demonstrated better executive functions in terms of inhibition [5,35] and better planning abilities [42] than children who did not engage in any physical activity. Studies conducted with children aged 8–9 years confirmed that sport influences changes in the right anterior prefrontal cortex, which are related to cognitive control [43]. The findings suggest, that plan-structured sport activities, for example tennis play, are associated with the development of inhibitory control. Although the development of inhibitory control and cognitive flexibility is slower in males than in females, the association between tennis play and inhibitory control and cognitive flexibility appears to be larger in males [44]. Additionally, playing football has a positive influence on executive functioning, including attention, in children [45]. In the case of attention, correlational and associational studies showed much weaker effects than studies which included interventions.

### 3.2. Thinking

“Thinking” is operationalized in this context as abstract thinking, conceptualizing cause-and-effect, creative thinking, and planning. Few studies have investigated the relation between physical activity and thinking. Children who participate in organized sport activities have been found to demonstrate a lower level of creativity as adults when compared to individuals participating in non-organized sport activities [46].

Apart from cross-sectional studies, a small number of studies with interventions were also conducted. Planning processes in children who attended a Football Exercise Program for at least six months were more developed in comparison to a control group of sedentary peers [45]. As in the case of attention, correlational and associational studies on thinking also showed much weaker effects than studies which included interventions.

### 3.3. Language

As in the case of attention and thinking, some of the previous research on the relationship between and influence of physical activity on speech was of a cross-sectional character and some involved an intervention. The results, independently of whether they show a relationship or an influence, indicate the positive role of physical activity in the development of speech.

Longitudinal research by Trudeau and Shephard [47] found a positive correlation between the number of hours devoted to sport and school grades. Children who devoted more time to sports were found to have significantly better grades [48]. Research by Carlson et al. [49] showed that girls who engage in sport for at least one hour per week had significantly better results in math and reading than girls who did not do at least one hour of sport. This relationship was not found to hold for boys in this study.

In studies involving interventions, a positive correlation has been demonstrated among German-speaking students when English lessons and sports activities were combined. This method of teaching improved the students’ English language grades [50]. Other studies highlight the positive impact of physical activity on the development of a broader lexical network and the comprehension of the meaning of words, as well as a greater ability to detect syntactic errors [34,35], and spelling performance [51]. In addition, sport was found to positively affect language understanding among primary school students [52].

Some authors report an association between physical activity and better grades at school [32,53]. Achieving better learning results is closely related to better executive functioning. Executive functions are of great importance for success in school and for the emotional development of children and adolescents [54]. They can be improved through both physical and cognitive training [54], such as computer training, games, or aerobics. According to some authors [55,56], aerobic activities have the most significant influence on executive functions, which control other cognitive functions [57,58,59]. Martial arts, yoga, and mindfulness training also stimulate the development of executive functions [60]. A training program can be called effective if it gradually increases the level of difficulty, leading to a satisfactory final effect [60]. Moreover, in order to achieve success in this area, one should develop the following aspects of executive functions: creativity, flexibility, self-control, and discipline.

As in the case of attention and thinking, correlational and associational studies on language and cognitive function also showed much weaker effects than studies which included interventions.

Changes in brain activity patterns associated with physical activity have also been observed in children [53]. Neuroimaging studies have revealed that 12-year-olds who were members of a dance group had higher somatosensory cortex activity [61]. However, in a study by Bunketorp Käll et al. [62] which was concerned with the hippocampal structures, no significant differences were observed. Some studies even indicate that physical activity before completing a task requiring decision making impacts more positively on its completion than passive activities (such as watching TV) before the task [25].

### 3.4. Learning and Memory

A decided majority of studies in this domain were based on different types of intervention trials. Previous research suggests that, overall, children who are more fit were found to have greater basal ganglia and hippocampus capacities [63]. These areas are associated with cognitive control and memory [43,63]. Among children aged 3–5 years, increased physical activity was found to improve their cognitive functions, especially in the area of working memory [64]. A similar improvement was observed in children who trained in karate [39]. A positive correlation was found between physical activity and better working memory among children aged 8–12 years [4,36,65,66]. Studies conducted by Kubesch et al. [37] demonstrated that the intensity of physical activity in children aged 12–14 years positively affected cognitive flexibility and operational memory. Similar results from Ishihara et al. [44] and Alesi et al. [45] found that tennis and football are associated with the development of working memory. In addition, physical activity is said to have a positive impact on visuospatial (V-S) memory [40,45]. Classroom-based physical activity (a 10-min bout of aerobic physical activity integrated with math practice) improved both physical activity levels and academic achievement. Results showed that among overweight children, physical activity improved performance in the Standard Flanker test by preventing the decline associated with seated practice [67]. Some results [68] suggest that game-based tennis lessons have beneficial effects on inhibitory control and physical fitness levels, and a longer duration of coordination training is associated with better working memory.

Research done by Mavilidi et al. [69] and Toumpaniari et al. [70] indicated that integrated physical exercises and gestures in preschool children achieved the best foreign language vocabulary learning outcomes.

### 3.5. Model

**Figure 2:**
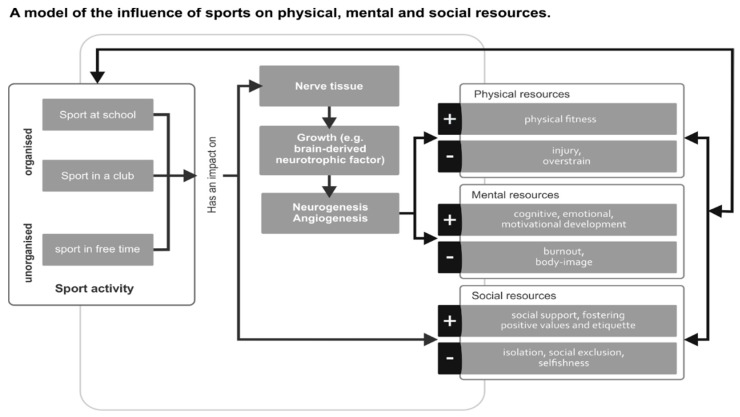
A model of the influence of sport on physical, mental, and social resources. The model was developed by the authors based on: McMorris, et al. [49], Diehl, et al. [71].

Taking the aforementioned results into consideration, most studies in this review indicate that physical activity is important for their physical resources (e.g., physical fitness, motor skills) of children in late childhood, for their mental resources (including cognitive functioning and executive functions, which are of special interest to us: motivation, ability to set goals, self-control, and emotional functioning), and for their social resources (e.g., social support, fostering positive values, and etiquette). These results are relevant independent of whether the physical activity is unorganized free play or organized activity, such as for a sports club and school activities. The following model, demonstrated in Figure 2, was developed to outline the influence of sports on physical, mental, and social resources.

## 4. Discussion

Key findings of most studies included in this review indicate that children’s engagement in physical activity may be associated with changes to certain brain structures, leading to an improvement in memory function (working memory in particular), as well as cognitive control. Independent of the children’s age category (early, mid, or late childhood), increased physical activity has been shown to improve cognitive function, especially in regard to working memory, V-S memory, and cognitive flexibility [36,37,39,65,66]. Moreover, research suggests that physical activity positively influences verbal functions, which facilitates the learning of words in a new language, leading to richer networks of words and their meanings, and also improves spelling performance, language understanding, and the detection of syntactic errors.

### 4.1. Late Childhood Period and Brain Development

The basic development of motor, cognitive, and social skills, which are crucial in further development, is already taking place in early and mid-childhood [72]. As such, studies that concern children in late childhood, whose executive functions are largely developed, are of special importance. The most intensive development of all components of executive functions, especially cognitive flexibility, happens at school age, usually between 7 and 12 years of age. Cognitive flexibility requires simultaneous inhibition of a dominant reaction, along with the remembering and activation of a new one, i.e.:

1. the efficient functioning of working memory, responsible for temporary storage of information to be processed, which contributes to the formation of complex cognitive functions, such as speech and operations on symbols; working memory assists in memorizing instructions and stages of plans of actions, as well as comparing alternatives; and
2. the efficient functioning of inhibition control, i.e., the ability to refrain from impulsive behavior, keep one’s attention focused, and pursue goals despite distractions, which also conditions stability and selectiveness of attention [73].

Late childhood (pre-adolescence) occurs at the age of 10–12 years. During this period, children undergo a number of biological, mental, and social changes [74,75,76]. The greatest development of frontal and temporal lobe gyri tends to occur before the age of 12 years [77]. The brain matures and develops very rapidly at this time, which in turn makes it particularly prone to environmental influences (both positive and negative). Recent research indicates that the increase of cognitive abilities of children and adolescents co-occurs with a decrease of grey matter density, which is caused by the loss of some synapses and the simultaneous strengthening of others [78]. Grey matter density peaks, and then decreases, approximately half way through the 11th year of age in girls (still in late-childhood), and approximately the 14th year of age in boys (already in adolescence) [78]. At the same time, white matter content in the brain increases, which is associated with cell myelination processes and increasing the efficiency of impulse conduction. This intense development and brain maturation also translates to limbic system functioning, which, in this period, is particularly sensitive to information from the environment [78]. It is worth noting that this is a period in which brain plasticity increases, which on one hand can help in seeking solutions to new challenges, but on the other, it is responsible for helplessness because it may underlie the sensitivity to all kinds of stress and toxic substances in the environment [79,80,81].

The synapses in the frontal cortex also become less dense in childhood. Moreover, there are changes in the electric activity of the brain, and an increase in the frequencies of fast brainwaves—around the age of 8–10 years, 6–9 Hz waves increase to 8–11 Hz [82]. Adaptation to changes in both the internal and the external environment is an ability that is possible due to the brain’s neuronal plasticity [78,83]. The brain’s plasticity allows an individual to acquire new skills and competences [78,83,84]. Researchers tend to agree that the highest plasticity of the brain occurs in childhood, and subsequently gradually decreases [84]. Brain development in pre-adolescence is 90% genetically determined. Gender differences between boys and girls have been reported with regards to the volumes of structures, such as the hippocampus or subcortical structures of white matter (e.g., corpus callosum), which are bigger in girls [78]. Functional activity studies show higher activity in the dorsolateral areas of the prefrontal cortex than among adults [83]. Pre-adolescence is associated with changes in brain structures and functioning in terms of executive functions, as well as rational memory [84,85]. Moreover, in late childhood, the level of hypothetical-deductive (formal) thinking increases, which allows for logical thinking and the forming of judgments [78,85].

### 4.2. Physical Activity in Childhood and Cognitive Functioning

In childhood, especially in late childhood, participation in physical activity is particularly important. A lack of physical activity in childhood can lead to limited perception and developmental disorders [86]. In addition, the period of late childhood is the time when motor skills develop the most dynamically [17], as well as cognitive functions, especially executive ones, which mature around the age of 12 years [87]. Executive functions allow one to engage in a situation through planning a given action, as well as to inhibit or postpone a given reaction [88,89]. Their efficient operation is associated with neuronal activity in the frontal lobes, especially in the dorsolateral prefrontal cortex, anterior cingulate cortex, parietal cortex, and subcortical structures, such as the thalamus, caudate nucleus, putamen, and cerebellum [88,89].

Physical exercise increases circulation, which leads to better oxygen supply to the brain, as well as providing the brain with nutrients [90,91,92]. Engaging in sports has a positive influence on all systems: the motor, cardiovascular, respiratory, hormonal, immunologic, and nervous systems. Thus, it stimulates the maturation of the motor areas in the brain, which in turn influences the motoric development and increases the speed of the conductance of nervous impulses [30,45,91,92,93,94]. Physical activity also stimulates the increase of neurohormonal secretion (substances produced by hypothalamic neurons and transported by blood or cerebrospinal fluid), having a significant impact on the excitability of neurons forming synapses [90]. School-age children who devote at least an hour each day to intensive physical activity show much better cognitive functioning, and researchers emphasize that, despite these unquestionable benefits, only about a third of children regularly engage in sports [95,96].

### 4.3. Limitations

The limitations of this review include the few available studies dedicated to the topic of interest, the small groups of participants taking part in these studies, a lack of cultural balance in these groups, and differences in methods, as well as the quality of reporting in the referenced studies. The number of high quality studies was relatively small. In these publications, results were reported in different formats (for example, raw scores, subscales, total scores). Several papers were identified and referenced as background literature; however, the majority of these studies duplicated work in the primary references and no additional studies were identified for potential inclusion in this review. Publications usually reported on only one component of cognitive functioning—e.g., only executive functions, attention, or memory. Indeed, there was a lack of comprehensive reports. Additionally, while some studies included information about the participants’ cognitive skills, many did not. Furthermore, there were no studies comparing children in different age groups. All publications referred only to early, mid, or late childhood. Finally, no studies explored how different types of physical activity or sports may differently influence children’s cognitive functioning.

Some of the conducted studies are of a cross-sectional character [34,35,38,39,42,44,46,52,61,63,68] and some include interventions of various kinds [4,5,15,16,31,32,37,40,41,43,45,50,51,57,60,62,64,65,66,67,69,70]. While this does not influence the direction of the results, it does impact things such as the effect size or the correlation coefficients. Notably, almost all studies indicate that the interventions (of various kinds, e.g., a short physical activity break, aerobic exercise, or afterschool physical activity program) are effective (with the exception of two studies, which do not confirm this positive influence of physical activity on cognitive functioning [31,32]), which is implied by the fact that the relationship between the physical activity and cognitive functioning (independently of its dimension) is stronger, and the effect size is larger. Both in cases of attention and thinking, as well as language, correlation/association with cognitive functioning were much weaker than the effect of interventions.

Due to the fact that too few studies use any particular type of intervention, it was impossible to make detailed comparisons between different types of interventions. Additionally, some researchers concentrated only on one domain of cognitive functioning, and others on a few selected ones or on all of them.

### 4.4. Implications for Research and Practice

Taking into account the limitations of the research to date, it would be worthwhile to conduct longitudinal studies of various, well-differentiated age groups (pre-school: 2/3–5/6 years, early school age: 8/9–11/12 years, early puberty: 11/12–14/14 years, and late puberty: 14/15–19/20 years). Furthermore, late childhood and adolescence constitute a sensitive period for cognitive development. As such, research should take care to appropriately distinguish various periods of adolescence, specifically late childhood, pre-adolescence, early adolescence, and adolescence. Well-organized measures are extremely helpful. Future research should also pay attention to the types of physical activity one engages in, and how different activities may differently influence cognitive functioning. Research should compare physically active groups with cognitively active groups—e.g., those engaging in various types of physical activity, and those playing musical instruments; not just those who are physically active compared to those with a sedentary lifestyle. Moreover, an optimal approach would be to take into account all cognitive functions in one study, instead of selecting only one, or a limited number.

Due to the small number of studies solely concerned with the influence of physical activity on the development of cognitive functions, it would be worthwhile to also undertake research focusing on how factors unique to sports influence the development of a child’s cognitive functions.

## 5. Conclusions

The literature indicates that efficient cognitive functioning in pre-adolescents requires not only an adequate intelligence quotient (IQ), but also high levels of executive function development (such as motivation, the ability to set goals, and self-control), which is fostered by engaging in sport. Of course, other activities undertaken by children, such as playing a musical instrument [45,97], are also associated with cognitive functioning, but physical activity, as the most natural for children of that age, is most desirable.

Results suggest that it is worthwhile to engage in sports in late childhood because it positively influences cognitive and emotional functions. Yet few studies have investigated the impact of sports on pre-adolescents’ cognitive functions or explored which cognitive functions are developed by which sporting disciplines. Such knowledge could be useful in developing training programs for preadolescents, aimed at improving cognitive functions important for a given sporting discipline.
